# Adversarial training with misaligned label correction for carotid segmentation from simultaneous non‐contrast angiography and intraplaque hemorrhage MRI

**DOI:** 10.1002/mp.17952

**Published:** 2025-07-15

**Authors:** Wenxu Zhang, Mingquan Lin, Kwok Leung Chan, Huijun Chen, Bernard Chiu

**Affiliations:** ^1^ Department of Electrical Engineering City University of Hong Kong China; ^2^ Department of Surgery University of Minnesota Minneapolis USA; ^3^ School of Biomedical Engineering, Tsinghua Medicine Tsinghua University Beijing China; ^4^ Department of Physics & Computer Science Wilfrid Laurier University Waterloo Canada

**Keywords:** adversarial learning, carotid vessel wall segmentation, noisy label, surrogate ground truth label

## Abstract

**Background:**

Simultaneous non‐contrast angiography and intraplaque hemorrhage (SNAP) imaging allows multi‐contrast MR images with large longitudinal coverage to be acquired in a single scan. With vessel wall boundaries available, vulnerable plaque components can be detected automatically from SNAP images. However, since SNAP imaging has not been previously used for vessel wall identification, vessel wall boundaries were required to be segmented from conventional multi‐contrast MRI first before registering to SNAP images. This registration process is not only time‐consuming but also prone to errors, potentially compromising subsequent plaque component analysis.

**Purpose:**

We aim to develop a model that directly segments the vessel wall from SNAP images, thereby eliminating the need for registration from another modality. The proposed model mitigates label noise arising from boundary misregistration.

**Methods:**

The proposed framework has a student‐mean teacher architecture, trained in two phases: (i) a warm‐up phase, in which the model was trained by well‐registered manual segmentations and minimizes Dice loss between predictions and manual labels and (ii) a fine‐tuning phase, in which the model was trained by both well‐registered and misaligned manual segmentations. This phase involves adversarial training with the fast gradient sign method (FGSM) and a novel surrogate label generator. The generator produced surrogate ground truth boundaries for each misaligned image by computing a weighted sum of the manual segmentation and the pseudo‐label, generated through selective hardening of predicted probabilities from the student and mean teacher models. The sum of the adversarial training loss and the Dice loss between the manual and predicted segmentations was minimized to obtain the final segmentation result. During inference, the averaged probability maps from the student and mean teacher models were used to assign voxels to their most probable class. This study utilized 129 image volumes (1474 axial slices), of which 74 volumes (810 axial slices) were well‐registered and 55 volumes (664 axial slices) were misaligned. Training involved 110 volumes (55 well‐registered and 55 misaligned), while validation and testing sets comprised 9 and 10 well‐registered volumes, respectively.

**Results:**

The proposed method outperformed existing noisy label learning methods when trained by the same set of misaligned segmentations. Results demonstrate our method's superiority with Dice similarity coefficient of 73.51±11.08%, 90.76±10.21%, and 90.10±10.38% for the vessel wall, lumen, and outer wall segmentations, respectively.

**Conclusion:**

The proposed segmentation framework effectively integrates noisy and reliable labels to produce accurate vessel wall segmentations directly from SNAP images. By eliminating the need for manual segmentation and inter‐modality registration, this approach facilitates more detailed plaque component analysis with reduced interslice distance across a longer arterial segment.

## 1 INTRODUCTION

Stroke is the second leading cause of death around the world.^1^ Carotid atherosclerosis is the main reason for approximately 7‐18% of all strokes.^2^ Therefore, a noninvasive imaging method for diagnosing potential ischemia and monitoring therapy progress is crucially needed. Although traditional multi‐contrast MR imaging techniques^1^ are capable of providing accurate characterization to plaque components without ionizing radiation, a long scanning time is required, and misregistration may occur between different weighting sequences.

To tackle these issues, the simultaneous non‐contrast angiography and intraplaque hemorrhage (SNAP) MR imaging method was proposed to acquire intrinsically registered multi‐contrast MR images to effectively monitor intraplaque hemorrhage and luminal stenosis,^3^ and Chen et al.^4^ showed that other high‐risk features, such as plaque calcification and carotid surface ulceration, can be detected using SNAP images. Zhang et al.^5^ demonstrated the feasibility of detecting vulnerable plaque components (including lipid‐rich/necrotic core and intraplaque hemorrhage) on SNAP images using machine learning classifiers. However, the vessel wall region, enclosed by the lumen and outer wall boundaries, was required for component classification. Since no validated approach has been developed for direct vessel wall segmentation from SNAP images, the approach used in Zhang et al.^5^ was to segment lumen and outer wall boundaries from conventional multi‐contrast images (T1‐weighted, T2‐weighted and time‐of‐flight [TOF] images) and then register the segmented boundaries to the SNAP image. There are two major limitations to this strategy. First, the longitudinal coverage of conventional multi‐contrast images is smaller than SNAP images and with a larger slice thickness. The need for vessel wall boundaries segmented from conventional multi‐contrast images limits the length of the artery that can be analyzed for plaque components. The larger slice thickness of the conventional multi‐contrast images also precludes a more detailed plaque component analysis with a smaller interslice distance. Second, the segmented slices from the conventional multi‐contrast images are required to be registered manually to the SNAP images; the manual registration takes time and introduces observer variability into the workflow. These limitations point to an important need for vessel wall segmentation directly from SNAP images.

However, before a direct vessel wall segmentation from SNAP images is developed and thoroughly validated, vessel wall boundaries from conventional multi‐contrast images are still required to be registered to SNAP images for training and validation of a direct SNAP segmentation model. Boundary misregistration can occur during this process due to manual misalignment and patient repositioning, thereby introducing label noise for segmentation model training. To the best of our knowledge, existing carotid artery segmentation methods rely on manual segmentation as the gold standard for training. Although using misaligned manual boundaries in training can compromise vessel wall segmentation accuracy, excluding all misaligned segmentations may result in a missed opportunity to develop a more generalizable segmentation model. We hypothesize that incorporating misaligned boundaries into the training process, alongside regularization and label correction techniques, can enhance segmentation performance compared to approaches that rely solely on well‐registered boundaries.

### 1.1 Related works

Deep learning models, particularly convolutional neural networks (CNNs), have been developed to segment carotid vessel wall from ultrasound and MR images.^6^, ^7^, ^8^ The effectiveness of these models heavily depends on access to large volumes of accurate ground truth data. However, in the presence of label noise, CNNs, due to their high capacity and large number of parameters, are prone to overfitting, which can significantly degrade performance.^9^ To mitigate this issue, researchers have introduced regularization techniques that improve the generalization ability of deep learning models and enhance their robustness against label noise.^9^, ^10^, ^11^, ^12^

Conventional regularization techniques, such as data augmentation,^12^ weight decay,^10^ dropout, and batch normalization,^9^ are shown effective in improving a model's resilience to label noise.^13^ For instance, Luo et al.^12^ showed that geometric transformations used in data augmentation can enhance a model's robustness to label noise. However, the effectiveness of conventional data augmentation methods was limited since pre‐defined operation sets can only provide finite combinations of transformations, which may not capture the full spectrum of variations that can occur in medical images.^14^

Adversarial training addresses this limitation by perturbing the correct label in the direction with which the model is most sensitive, thereby providing the maximum regularization effect. Adversarial training for deep learning model originates from Goodfellow et al.,^15^ in which they found that deep neural network was extremely sensitive to gradient‐based attack; adding invisible adversarial noise into the input image could significantly deviate a well‐trained model from the original predictions. Inspired by this observation, fast gradient sign method (FGSM) was proposed to approximate the adversarial perturbations, in which regularization was achieved by supervising the model's predictions for perturbed images. Arpit et al.^10^ showed that combining adversarial training with dropout was the best approach in increasing the model's robustness to noisy labels, as compared to other regularization methods, including weight decay, dropout alone, and input Gaussian noise. However, directly deploying adversarial training on noisy labels can lead to sub‐optimal performance since adversarial perturbation computed from the incorrect label may not represent the direction that could deviate the model's prediction from the true label.^16^ Training the model with such perturbed noisy labels would deteriorate the model's performance. Therefore, a method that could correct labels for adversarial training can enhance the model performance.

**FIGURE 1:**
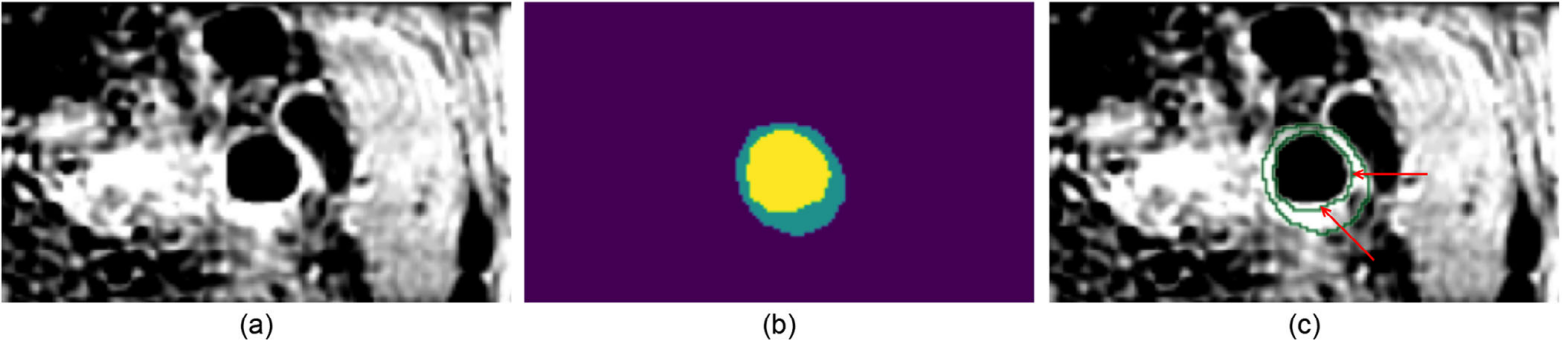
An example SNAP image with misaligned segmentation. (a): CR image; (b): manual segmentation; (c) contours of vessel wall segmentation (in green color) superimposed to the CR image with misalignment highlighted by red arrows (best view in color). CR, phase‐sensitive reconstruction; SNAP, simultaneous non‐contrast angiography and intraplaque hemorrhage.

Typical approaches for correcting noisy labels involve pseudo‐labels derived from the model's predictions.^17^, ^18^, ^19^ Pseudo‐labeling is achieved by selecting the class with the highest probability from the model's predictions. Pseudo‐labeling leverages the insight that deep learning models tend to gain confidence in their predictions while being trained on correctly labeled data. Consequently, incorrect labels can often be identified by their discrepancy from the model's predictions.^18^, ^20^ Tanaka et al.^17^ demonstrated that replacing noisy labels with pseudo‐labels for loss supervision could improve the model's performance on the noisy label set. However, this approach discarded the useful information hidden in the noisy labels. In our application, the misaligned segmentations should not be discarded, since misalignment only occurred in a portion of the manually segmented contour, as shown in Figure 1, and can still contribute positively to the segmentation performance. Reed et al.^18^ preserved the useful information in noisy labels by computing a convex combination of pseudo‐labels and noisy labels, which serves as surrogate labels for training. Inspired by this approach, we adopted a similar strategy to generate surrogate labels for our training process. However, the quality of their surrogate labels relies heavily on the converging state of the model, which can be unstable since the gradient would fluctuate during training.^21^, ^22^ Such instability could affect the quality of the surrogate labels, thereby compromising the model's performance.

The mean teacher model was proposed to stabilize the training process by generating steadily evolving predictions as fitting targets.^23^, ^24^ Nguyen et al.^22^ employed this model during training and ensembled its predictions from different epochs to identify and remove the noisy labels. Although we do not intend to remove noisy labels as in their work, their results suggested that the mean teacher model can be used to generate stable pseudo‐labels, and therefore, surrogate labels in our framework.

### 1.2 Contributions

In this study, a segmentation framework is proposed to enhance the model's robustness to the label noise stemming from the misaligned segmentations. The central hypothesis of this work is that, through the introduction of regularization and label noise correction strategies, the segmentation performance of a deep learning model can be improved by additional training with SNAP images equipped with misaligned segmentations. The proposed robust learning framework leverages FGSM to perform adversarial training on the student segmentation model. FGSM approximates adversarial perturbations with the strongest effect in deviating the model prediction from the correct class, thereby reducing the chance for the model to overfit misaligned segmentations.

In addition, a surrogate label generator was designed to generate surrogate segmentations as the fitting target for adversarial training. We propose a surrogate label generation approach that creates a stable pseudo‐label by ensembling the predictions from the student and mean teacher branches, followed by a probability “hardening” process. The probability hardening strategy boosts the class prediction probability to 1 for a pixel when both the student and mean teacher branches make confident predictions for the same class. The hardened pseudo‐label, instead of the misaligned manual segmentations, was used as the ground truth for regularization. We show that our model outperforms existing noisy label learning methods in segmenting the lumen, outer wall and vessel wall of carotid arteries in SNAP images.

## 2 METHOD

### 2.1 Data acquisition and preprocessing

In total, SNAP and conventional multi‐contrast images were acquired from 68 patients (44 female, 24 male) aged from 49 to 67 years using a whole body 3.0T MR scanner (Achieva TX, Philips Healthcare, Best, The Netherlands), as described in Zhang et al.^5^ The study protocol was approved by the Institution Review Board and all participants provided written informed consent.^5^ SNAP acquisition was performed in the coronal plane and covered the whole carotid artery and part of intracranial arteries (250 mm in head‐feet direction) and required about 7 min (voxel spacing = 0.8 ×0.8× 0.8 mm^3^). MR acquisition using the conventional multi‐contrast imaging protocol, which included three‐dimensional TOF, two‐dimensional quadruple inversion recovery (IR) T1‐weighted imaging, and two‐dimensional double IR T2‐weighted imaging, was conducted on the same scanner, covering approximately 48 mm of the carotid artery in about 12 min. The in‐plane resolution was 0.6 × 0.6 mm^2^ for all the conventional multi‐contrast sequences, while the slice thickness was 2 mm for T1‐ and T2‐weighted sequences, and 1 mm for the TOF sequence.

The IR and the reference (REF) images were obtained through two successive gradient echo acquisitions in the SNAP sequence.^3^ The phase‐sensitive reconstruction (CR) image was then generated by correcting the background phase of the IR acquisition based on the REF image.^3^

**FIGURE 2:**
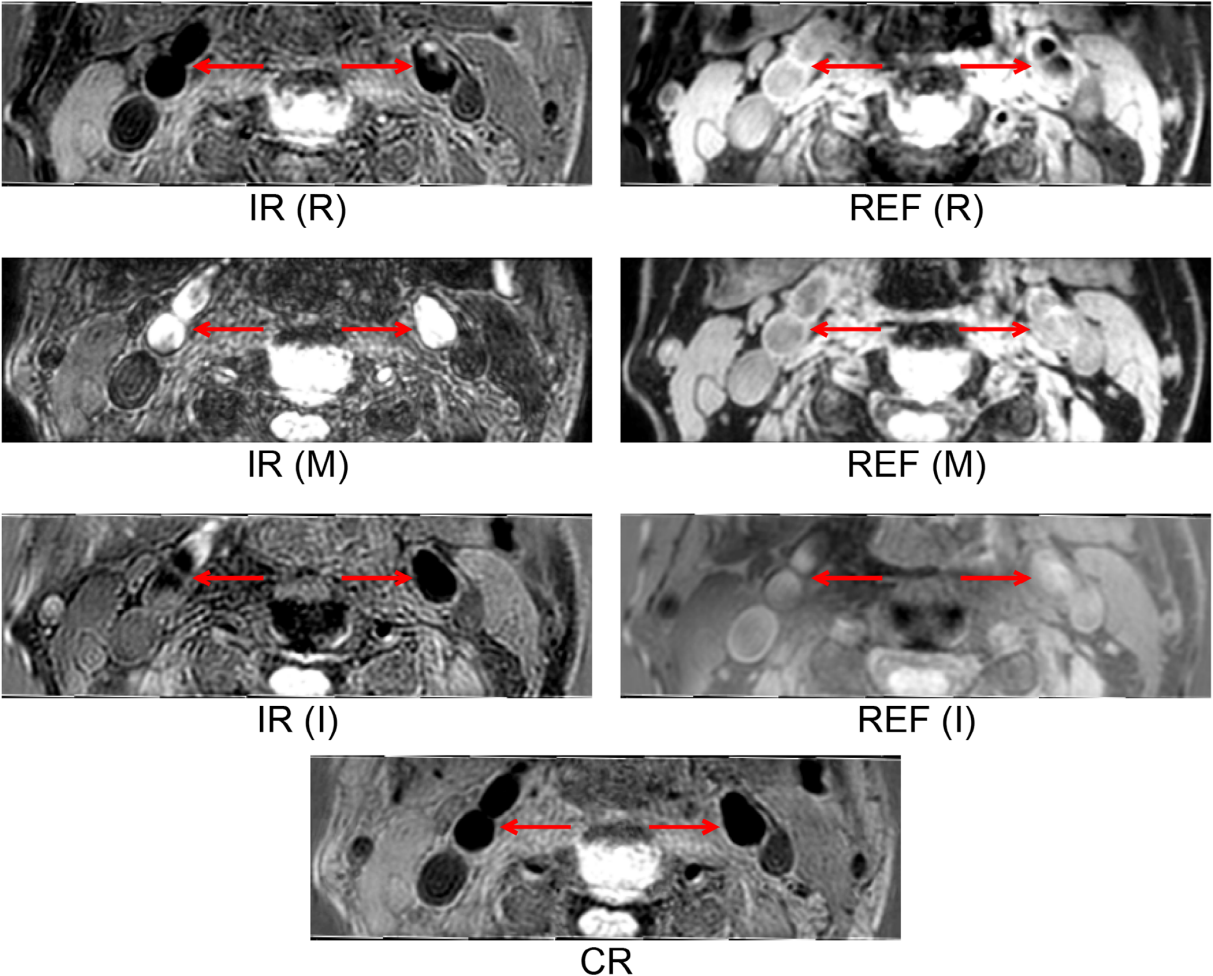
Visualization of the resliced SNAP multiple contrast images, in which the left and right carotid arteries were indicated by red arrows. IR, REF and CR denote the inversion recovery, reference and phase‐sensitive reconstruction images from the SNAP sequence, respectively. M, R and I enclosed by brackets refer to magnitude, real and imaginary parts, respectively. SNAP, simultaneous non‐contrast angiography and intraplaque hemorrhage.

In addition to the magnitude representation of the IR and REF images, their real and imaginary representations provide additional intensity features. In total, seven image sets were generated from the SNAP sequence. Figure 2 shows the seven SNAP images acquired for an example subject at the same axial location.

To generate the ground truth for vessel wall contours for the SNAP images, the lumen and the outer wall were first segmented by two experienced radiologists independently from the conventional multi‐contrast images. As the slice thicknesses of the T1‐ and T2‐weighted images are 2mm, the interslice distance between adjacent contours is 2mm.^5^ Then, the original 3D SNAP images were resliced to match the thickness and the spatial location of the conventional multi‐contrast images. A third reviewer then mapped the contours segmented on the conventional multi‐contrast images to the resliced 3D SNAP images.

Following the protocol used for multi‐contrast MRI analysis,^25^ the conventional multi‐contrast MR images were processed by a zero‐filled Fourier Transform operation to reduce the in‐plane pixel size to 0.31 × 0.31 mm^2^ for better visualization of small plaque structures. The SNAP images were interpolated to match this pixel size to allow for accurate registration and improved visualization.

The image quality of the SNAP images and the registration accuracy from the conventional multi‐contrast to the SNAP images were manually rated according to the criteria described in Zhang et al.^5^ Specifically, qualitative assessment was conducted on the lumen delineation for each CR image on a slice‐wise basis. The SNAP image slice was considered to have good image quality if less than half of the lumen boundary in the CR image was obscure or invisible. Axial SNAP images were otherwise rated as having poor image quality. A SNAP image volume was analyzed only if at least 8 consecutive axial slices in the volume were rated as good quality. Meanwhile, SNAP image slice was considered well‐registered if the contours segmented from the conventional multi‐contrast sequence could match the carotid artery in the SNAP image and it is otherwise considered as misaligned. An image volume was considered well‐registered if all its image slices were well‐registered.

The 3D SNAP image volume acquired from each patient was cut through the medial sagittal axis into two images, one enclosing the left and the other the right carotid artery. Out of the 136 SNAP image volumes available (68 patients, left and right arteries), 7 were not analyzed because they have fewer than 8 consecutive slices rated as good quality. Axial images in the remaining image volumes were down‐sampled to a resolution of 224 × 224 voxels, followed by normalization to zero mean and unit variance for images of each contrast. As a result, 129 image volumes (1474 axial slices) were involved in our analysis, comprising 55 misaligned volumes (664 axial slices) and 74 well‐registered volumes (810 axial slices). Patient‐based grouping was used for partitioning training, validation and testing sets to ensure the independence of different partitions. In total, 9 and 10 well‐registered image volumes (108 and 131 slices, respectively) were allocated to the validation, and testing sets, respectively. The remaining 55 well‐registered image volumes (571 axial slices) were combined with the 55 misaligned image volumes to form the training set, consisting of 110 SNAP image volumes (1235 axial slices) in total.

### 2.2 Overview of the proposed learning framework

**FIGURE 3:**
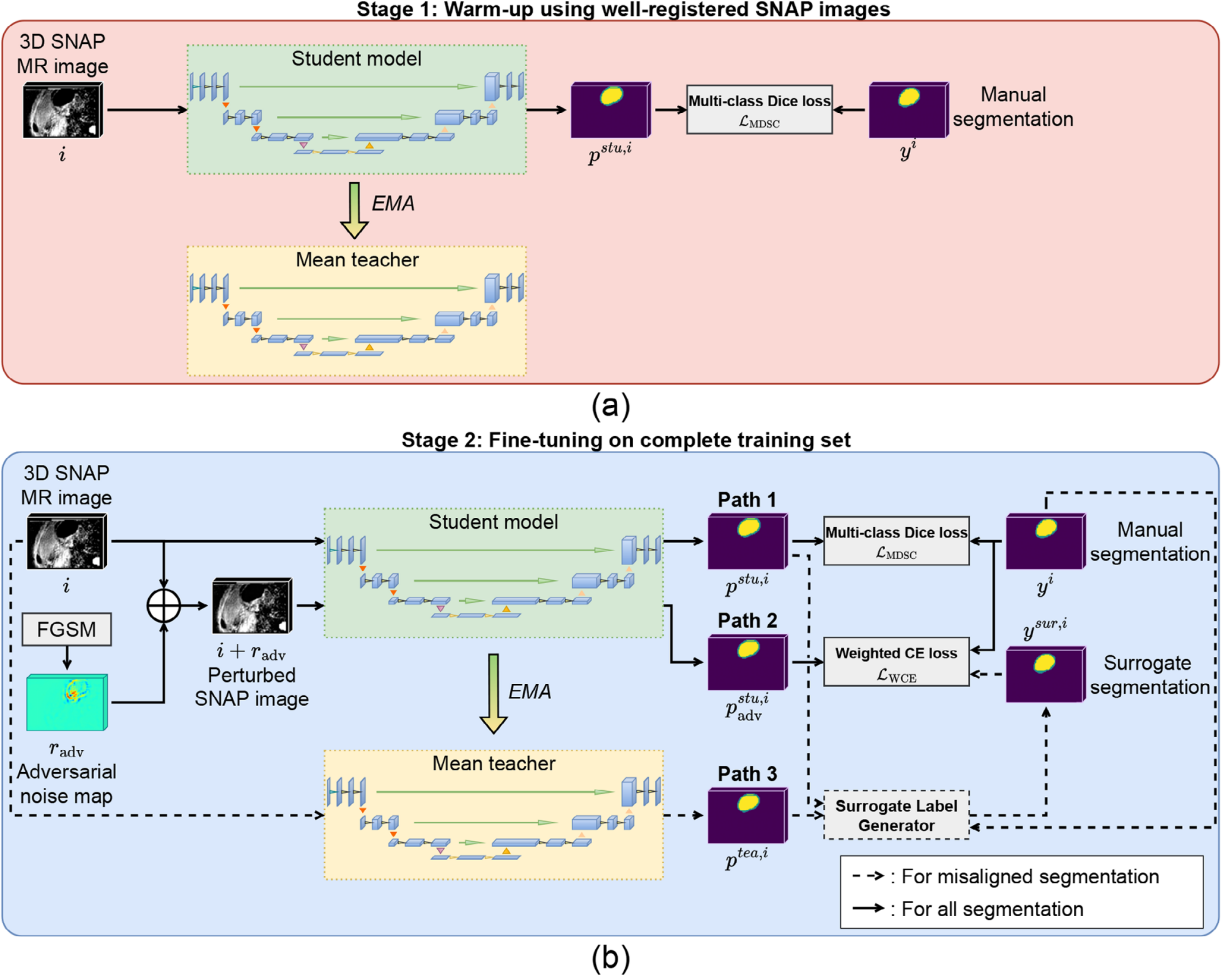
Overall framework of the proposed learning framework consisting of (a) the warm‐up stage involving only well‐registered images and (b) the fine‐tuning stage involving both well‐registered and misaligned images.

Figure 3 shows the overall structure of the proposed learning framework, which involves two modified 3D U‐Nets described in the Appendix to serve as the student and mean teacher models. The student model's parameters were optimized by the loss functions used in the framework, whereas the mean teacher model's parameters were updated from the student model using the exponential moving averaging (EMA) throughout training. A two‐stage learning strategy is proposed, which consists of a warm‐up phase trained only by images with the well‐registered segmentation and a fine‐tuning phase involving the entire training set comprising all well‐registered and misaligned images.

Figure 3a shows the workflow of the warm‐up stage, in which well‐registered SNAP images were used to train the student model. Although the mean teacher model was not involved at this stage, its parameters were updated by EMA based on the student model's parameters. Figure 3b illustrates the workflow of the fine‐tuning stage, where in addition to training with the manual segmentation (Path 1), the perturbed SNAP images were incorporated as an additional branch (Path 2) to regularize the student model. For images with misaligned boundaries, this regularization branch was trained with a surrogate segmentation, created by summing the manual segmentation with the hardened, ensembled prediction from the student (Path 1) and mean teacher models (Path 3). At inference, the predicted segmentation was produced by averaging the student and the mean teacher segmentations. The following provides details on the segmentation backbone, the proposed two‐stage learning strategy and the inference procedure.

### 2.3 Warm‐up using well‐registered segmentations

The proposed approach starts with warm‐up training as successfully employed by previous methods involving noisy labels.^26^, ^27^ As shown in Figure 3a, the student model was trained by well‐registered segmentations at the warm‐up stage and the training process was driven by the multi‐class Dice loss function, which has been shown effective in detecting small targets and has been used in carotid segmentation.^6^, ^8^ Each 3D SNAP image volume $i\in R^{N\times H\times W\times 7}$ corresponds to its manually segmented boundaries $y^{i}\in {\left\{0,1\right\}}^{N\times H\times W\times 3}$, represented in a three‐channel one‐hot encoding format, in which the three channels represent voxels enclosed by (1) lumen (2) vessel wall and (3) background. N, H and W represent the number of axial slices in the image volume and the height and width of each image slice, respectively. The seven channels of the image volume i correspond to the seven modalities generated from the SNAP sequence. The multi‐class Dice loss is the mean Dice loss associated with the lumen and vessel wall boundaries, as defined as follows:

(1) $$\begin{array}{cc} & L_{\text{MDSC}}\left(p^{stu,i},y^{i}\right)\\ & \;=\frac{L_{\text{DSC}}\left(p_{LM}^{stu,i},y_{LM}^{i}\right)+L_{\text{DSC}}\left(p_{VW}^{stu,i},y_{VW}^{i}\right)}{2}, \end{array}$$

where $y_{LM}^{i}$ and $y_{VW}^{i}$ correspond to the manual segmentations of the lumen and vessel wall, respectively. $p_{LM}^{stu,i}$ and $p_{VW}^{stu,i}$ represent the probability maps generated by the model for the lumen and vessel wall, respectively. $L_{\text{DSC}}$ is the binary Dice loss, defined as $L_{\text{DSC}}\left(x,\hat{x}\right)=1-\frac{2\left|x\times \hat{x}\right|}{\left|x+\hat{x}\right|}$, where + and × represent element‐by‐element addition and multiplication, respectively.

Although the student model is being trained, the mean teacher model was updated based on EMA:

(2) $$\theta_{j}^{t}=\alpha \theta_{j-1}^{t}+(1-\alpha )\theta_{j}^{s},$$

where $\theta_{j}^{s}$ denotes the parameters of the student model at Iteration j, $\theta_{j-1}^{t}$ denotes the parameters of the mean teacher model at Iteration j−1 and α denotes the smoothing coefficient.

### 2.4 Fine‐tuning on complete training set

The mean teacher models were trained in the fine‐tuning stage by the complete training set consisting of both the well‐registered and misaligned SNAP images. As shown in Figure 3b, the fine‐tuning stage involves three branches. Path 1 involves the student model prediction from the original SNAP image. Path 2 involves the student model prediction from the perturbed SNAP images generated by adding adversarial noise to the original image, as detailed in Section 2.4.2 Path 3 involves the mean teacher model prediction from the original SNAP image. The predictions in Paths 1 and 3 were combined to generate the surrogate ground truth label to train the segmentation in misaligned images, as described in Section 2.4.1 The student model was jointly supervised by the multi‐class Dice loss through Path 1 and the weighted cross‐entropy (CE) loss through Path 2 with the mean teacher parameters updated based on EMA as in Equation (2).

#### 2.4.1 Surrogate label generator

**FIGURE 4:**
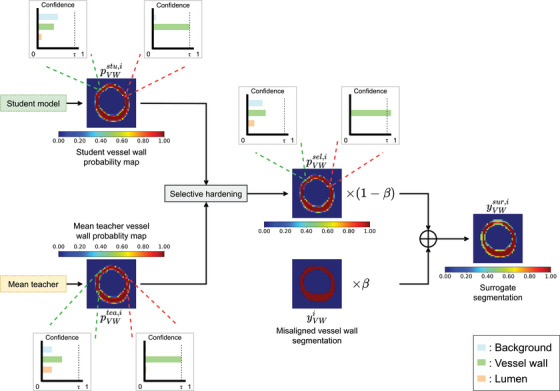
Illustration of the surrogate label generation process with two example voxel. The voxels on the left and the right are pointed to by the green and red dotted lines, respectively. The probability maps for the vessel wall class were colour‐coded and shown here. Hardening was performed for the voxel on the right but not the voxel on the left. τ is the minimum probability for hardening to be performed. β controls the weights of the pseudo‐label and manual segmentation in generating the surrogate ground truth used to train the adversarial path (Path 2 in Figure 3).

The surrogate label was generated for each misaligned boundary. The workflow of the surrogate label generation process is illustrated in Figure 4, which involves generating a selectively hardened pseudo‐label based on the predictions from Paths 1 and 3. Although label hardening has shown effective in improving model's classification performance from noisy labels,^18^ hardening all voxels in the predicted segmentation would strengthen model noise, thereby lowering the model performance. To address this issue, only voxels segmented with “high confidence” were hardened. Voxels satisfying the following two conditions were considered as those segmented with high confidence: (1) the student and mean teacher models both predict the same class with the highest probability and (2) this class has a probability that exceeds a target threshold in both the student and mean teacher's predictions.

Specifically, suppose $p^{tea,i,k}$ and $p^{stu,i,k}$ are three‐dimensional vectors denoting the probabilities for lumen, vessel wall and background at voxel k generated by the mean teacher and the student model for Image i, respectively. Suppose u is the channel index of the most confident class in $p^{tea,i,k}$ (i.e., $p^{tea,i,k}\left(u\right)=\max_{n\in \{0,1,2\}}p^{tea,i,k}\left(n\right)$). Similarly, $p^{stu,i,k}\left(v\right)=\max_{n\in \{0,1,2\}}p^{stu,i,k}\left(n\right)$. The probability vector associated with voxel k of the selectively hardened pseudo‐label, denoted by $p^{sel,i,k}$, is expressed by:

(3) $$\begin{array}{cc} & p^{sel,i,k}\\ & =\left\{\begin{array}{cc} 1_{u}, & \text{if}\;u=v,\;p^{tea,i,k}\left(u\right)>\tau ,\;p^{stu,i,k}\left(v\right)>\tau .\\ \frac{1}{2}\left(p^{tea,i,k}+p^{stu,i,k}\right), & \text{otherwise.} \end{array}\right. \end{array}$$

where $1_{u}$ denotes a three‐channel one‐hot encoded pseudo‐label vector, in which only the value at channel u equals to 1 and the other two values are 0 and τ is the target threshold for selective hardening. We note that Equation (3) indicates that hardening was only applied in the first scenario. Figure 4 shows an example voxel located inside the vessel wall with hardening applied (pointed to by red dotted lines) and an example voxel located at the border of the outer wall without hardening (pointed to by green dotted lines). The surrogate ground truth label $y^{sur,i}\in {\left[0,1\right]}^{N\times H\times W\times 3}$ for a misaligned axial image was computed as the weighted sum of the manual segmentation $y^{i}$ and the selectively hardened pseudo‐label $p^{sel,i}\in {\left[0,1\right]}^{N\times H\times W\times 3}$ as follows:

(4) $$y^{sur,i}=\beta y^{i}+\left(1-\beta \right)p^{sel,i}.$$

where β is the weighting constant.

#### 2.4.2 Adversarial training

FGSM^15^ was used to compute the adversarial noise map approximating an ideal adversarial attack. Let $i\in R^{N\times H\times W\times 7}$ denotes the input image, ${\tilde{y}}^{i}$ represents the fitting target of Image i and $p(i,\theta^{s})$ represents the probability map predicted by the student model for Image i. The ideal adversarial attack $r_{\text{adv}}\in R^{N\times H\times W\times 7}$ for Image i is approximated by:

(5) $$r_{\text{adv}}\approx \varepsilon \text{sign}\left(\nabla_{i}D\left(p\left(i,\theta^{s}\right),{\tilde{y}}^{i}\right)\right).$$

where $\nabla_{i}$ represents the gradient operator with respect to the image, D(·,·) denotes the CE operation and ε controls the strength of adversarial noise.

The training of the student model through Path 2 was based on the weighted CE loss between the segmented mask and ${\tilde{y}}^{i}$, obtained by first computing the weighted CE loss value on each voxel, then taking the average of all voxels as in Goodfellow et al.^15^ In our study, we assigned higher weights to the lumen and vessel wall channels than the background channel to mitigate the class imbalance between foreground and background. With the segmented mask denoted by $p_{\text{adv}}^{stu,i}$ as in Figure 3b, the loss in Path 2 is expressed by:

(6) $$\begin{array}{cc} & L_{\text{WCE}}\left(p_{\text{adv}}^{stu,i},{\tilde{y}}^{i}\right)\\ & \;=\frac{1}{HWN}\sum_{k=1}^{HWN}\sum_{j=1}^{3}-w\left(j\right){\tilde{y}}^{i,k}\left(j\right)\log\left(p_{\text{adv}}^{stu,i,k}\left(j\right)\right), \end{array}$$

where ${\tilde{y}}^{i,k}$ represent the three‐channel one‐hot encoded manual segmentation at pixel k, with j representing the channel index. The three‐channel weight vector w assigns importance to each class, with values set to 2, 2, and 1 for the lumen, vessel wall, and background, respectively. The overall loss function for the fine‐tuning stage is the summation of the losses associated with Paths 1 and 2:

(7) $$L_{\text{all}}=L_{\text{MDSC}}\left(p^{stu,i},y^{i}\right)+L_{\text{WCE}}\left(p_{\text{adv}}^{stu,i},{\tilde{y}}^{i}\right).$$

where $y_{i}$ is the manual segmentation. ${\tilde{y}}^{i}$ is the manual segmentation for well‐registered images and the surrogate ground truth for misaligned images. $p^{stu,i}$ and $p_{\text{adv}}^{stu,i}$ are the segmentation probability maps from Paths 1 and 2, respectively.

### 2.5 Inference

**FIGURE A.1:**
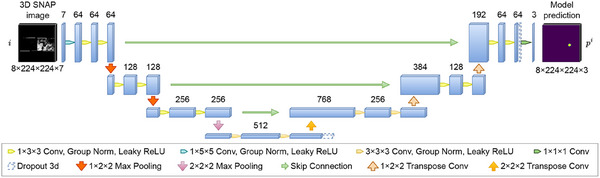
Architecture of the modified 3D U‐Net. Group Norm: Group normalization; ReLU: Rectified linear unit.

Algorithm segmentation was generated by first averaging the probability maps produced by the student and mean teacher models and then categorizing each voxel to the class with the largest average probability. Although the number of slices in a 3D SNAP image volume varies from 8 to 16, the model was implemented to segment 8 slices per volume, as shown in Figure A.1. For input image volumes with more than eight slices, the first and last eight slices were separately fed into the segmentation model.

**FIGURE 5:**
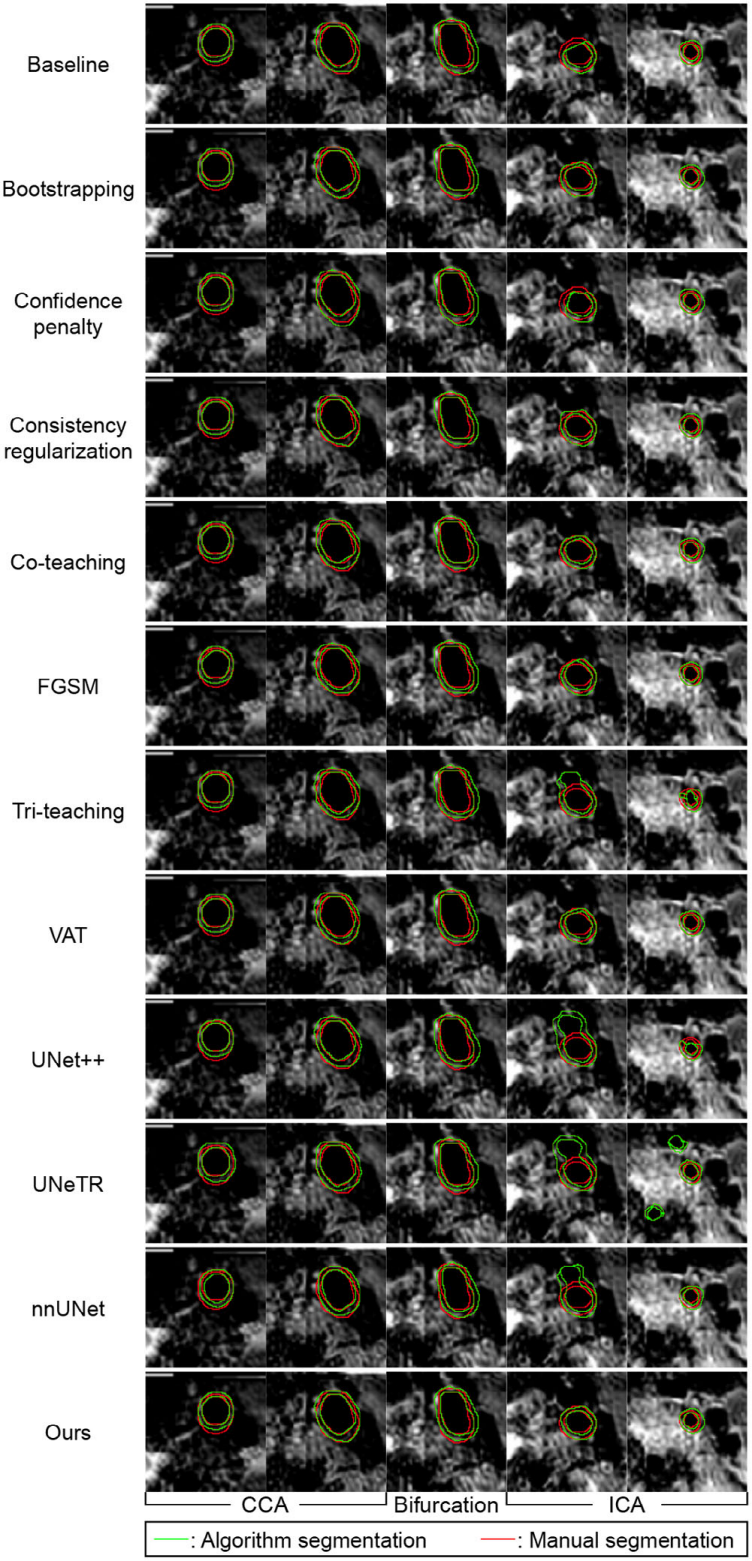
Visualization of manually segmented (red) and predicted (green) contours superimposed on the CR images of an example artery. The axial image sequence consists of two CCA images (one located at the most proximal to bifurcation and one at the most distal), one bifurcation image and two ICA images (positioned at the most proximal and distal locations relative to the bifurcation). CR, phase‐sensitive reconstruction; CCA, common carotid artery; ICA, internal carotid artery.

**FIGURE 6:**
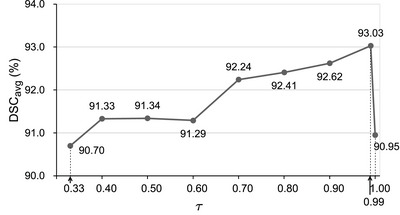
$DSC_{avg}$ of the proposed method in the validation set with different confidence level threshold τ. DSC, Dice similarity coefficient.

For image volumes with fewer than 16 slices, there are overlapping slices that were segmented twice. For these slices, the final segmentation mask was determined by averaging the two probability maps and assigning each voxel to the class with the highest average probability.

### 2.6 Experiments

#### 2.6.1 Evaluation metrics and statistical analysis

The Dice similarity coefficients (DSC), 95% Hausdorff distance (HD95) and average surface distance (ASD) were used to evaluate segmentation accuracy. Statistical analysis using Tukey's Honesty Significance Difference (HSD) test was performed to compare the difference in DSC attained by different segmentation models,^28^ with the difference considered statistically significant if the probability of making a type I error was less than 5% (*p* < 0.05).

#### 2.6.2 Data augmentation

During training, SNAP images were augmented on‐the‐fly to prevent overfitting. First, for SNAP image with more than eight slices, the corresponding training image was formed by random sampling eight consecutive slices from the original SNAP image to improved the variation of training set. Therefore, the size of each training image was fixed as 8 ×224× 224 (number of slices, slice height and slice width) in our experiments. Then, augmentation composed of the following operations was applied to training image in a slice‐wise manner: random rotation with angles of {0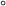, 90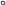, 180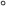, and 270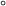}, random contrast with an enhancement factor ranging [0.8, 1.2], random gamma correction with range of [0.8, 1.2] and random horizontal flipping. We note that the augmentation operations performed on slices of the same image volumes were kept the same to maintain the longitudinal continuity of the SNAP images. The four angles were chosen for random rotation to avoid the need for resizing or cropping operations to preserve image dimensions.

The proposed model and methods reproduced in our comparison were all trained with 8000 iterations, with an iteration defined as training the model with a mini‐batch, except for the nnUNet that specified its optimal training schedule in the original implementation.^29^ For the proposed method, 4000 iterations were run for each of the warm‐up and fine‐tuning phases. The training batch size was set as 4, and Adam optimizer^30^ with weight decay of $5\times 10^{-4}$ was used when training all methods. The learning rate was fixed at $2\times 10^{-4}$ at the first 4000 iterations and was multiplied by 0.9 for every 500 iterations until the training was completed. The model was optimized based on the validation set. The lumen and outer wall segmentations were generated for the validation set in the fine‐tuning phase as described in Section 2.5. The mean DSC for the lumen and the outer wall segmentations (denoted by $DSC_{avg}$) were computed with the model attaining the highest $DSC_{avg}$ applied to segment test images. All methods were implemented using PyTorch framework and trained using two NVIDIA GeForce RTX 3090 GPUs.

## 3 RESULTS

### 3.1 Hyperparameter tuning

The proposed framework has four hyperparameters: the EMA smoothing coefficient α (Equation 2), the weight of manual segmentation in the generation of the surrogate label β (Equation 4), the confidence threshold for pseudo‐label hardening τ (Equation 3), and the strength of adversarial perturbation ε (Equation 5). These hyperparameters were initialized empirically and tuned by sequential optimization on the validation set with $DSC_{avg}$ serving as the evaluation metric for model performance.

After tuning each hyperparameter, the values of the hyperparameters previously tuned were held at their optimized values while the next hyperparameter was being tuned.

This tuning procedure resulted in the following optimized hyperparameters: α=0.99, β=0.5, τ=0.99 and ε=4, which were used in the remaining experiments.

### 3.2 Ablation study

The proposed learning framework consisted of two innovative components: (i) adversarial training and (ii) the integration of the manual segmentation and the pseudo‐labels from the student and mean teacher models to generate an improved reference for misaligned images in adversarial training. The following ablation experiments were designed to evaluate the individual contribution of the above two components.

The baseline model involves only the student network trained with the multi‐class Dice loss generated by comparison. The training of the baseline model involves both the warm‐up and the fine‐tuning phases. Model I adds the adversarial training branch (Path 2 in Figure 3) to the baseline, with the fitting target set to be the manual segmentation to disable the surrogate labels. Model II adds the mean teacher network to Model I. The difference between Model I and II is the inference in Model II was made by averaging the results from the student and mean teacher models, instead of just using the result from the student model as in Model I. Model III adds the surrogate label generator to Model II but does not involve the label selective hardening step. In Model III, the surrogate ground truth is the weighted sum of manual segmentation and the average prediction of student and mean teacher models. The full model adds the selective hardening to Model III.

Table 1 reports the results generated by the five models. The DSC improvement contributed by adversarial training was statistically significant for vessel and outer wall segmentations, as demonstrated by the comparison between Model I and the baseline (Vessel wall: $p=4.6\times 10^{-2}$; Lumen: $p=7.5\times 10^{-2}$; Outer wall: $p=4.5\times 10^{-2}$). There is no significant difference in the DSCs between Model I and II (Vessel wall: p=0.91; Lumen: p=0.91; Outer wall: p=0.93). The surrogate label generator without selective hardening has improved the DSCs as compared to Model II, but the improvements are not statistically significant (Vessel wall: p=0.61; Lumen: p=0.67; Outer wall: p=0.63). Selective hardening has provided statistically significant improvement to the DSCs in vessel wall and outer wall, as demonstrated by the statistical comparison between Model III and the full model (Vessel wall: p=0.023; Lumen: p=0.059; Outer wall: p=0.037), but the DSC improvement in lumen was close to but not statistically significant.

**TABLE 1:** Results of the ablation studies.

	Components				DSC (%)		
Models	Adversarial training	Mean teacher	Surrogate label generation w/o selective hardening	Surrogate label generation with selective hardening	Vessel wall	Lumen	Outer wall
Baseline					63.03±25.73	79.98±28.26	78.72±29.18
I	✓				68.54±17.69	85.43±20.23	84.94±19.73
II	✓	✓			68.30±16.40	85.70±18.70	84.74±18.5
III	✓	✓	✓		69.37±17.58	86.77±21.67	85.90±20.30
Full	✓	✓		✓	**73.51** ± **11.08**	**90.76** ± **10.21**	**90.10** ± **10.38**

*Note*: The symbol ✓ indicates a specific component was activated in a model. The highest DSCs are highlighted in bold.

Abbreviation: DSC, Dice similarity coefficients.

An important hypothesis of our study was that incorporating misaligned boundaries into the training process would boost segmentation performance. To test this hypothesis, we remove all misaligned images from the training set and train the full model with a dataset that only consists of well‐aligned images. As misaligned boundaries were not involved, there was no need to generate the surrogate segmentation label. The DSC for the vessel wall, lumen and outer wall attained in this experimental setting are 65.26±20.32%, 81.21±25.07% and 80.98±24.43%, respectively, which are statistically significantly lower than the full model with misaligned boundaries included in the training set (Vessel wall: $p=6.2\times 10^{-5}$; Lumen: $p=7.6\times 10^{-5}$; Outer wall: $p=1.15\times 10^{-4}$).

### 3.3 Comparison with existing methods

The segmentation performance of the proposed method was compared with existing methods for handling noisy labels: bootstrapping‐hard,^18^ confidence penalty,^11^ consistency regularization,^12^ co‐teaching,^31^ Tri‐teaching,^32^ FGSM,^15^ and VAT.^33^ For a fair comparison, all noisy label learning approaches were trained with the same backbone model, the same two‐stage training policy (i.e., warm‐up and fine‐tuning) and the same number of iterations. The $DSC_{avg}$ was optimized in all methods based on the validation set. Co‐teaching and tri‐teaching involve multiple models just as our method. For these models, the $DSC_{avg}$ were computed by averaging the segmentation results of the sub‐models. Additionally, the proposed method was compared with three state‐of‐the‐art deep learning models: UNeTR,^35^ UNet++,^34^ and nnUNet.^29^ The implementation of the three models was provided by their respective authors on the open source platform. In this comparison, UNeTR and UNet++ were trained with the multi‐class Dice loss, the same two‐stage training policy (i.e., warm‐up and fine‐tuning) and the same number of iterations as our method. nnUNet was trained with the schedule provided by the official implementation. Since the carotid arteries were segmented from SNAP image volumes in this study, the three‐dimensional version of UNeTR, UNet++ and nnUNet were evaluated.

Table 2 compares the DSC, HD95, and ASD measurements attained by the proposed method with those of existing noisy label learning approaches and the state‐of‐the‐art segmentation models. The DSCs of our model are statistically higher than all noisy label learning methods (Vessel wall: $8.0\times 10^{-6}\leq p\leq 2.9\times 10^{-3}$; Lumen: $5.8\times 10^{-5}\leq p\leq 1.2\times 10^{-2}$; Outer wall: $3.8\times 10^{-5}\leq p\leq 8.8\times 10^{-3}$), as well as the three state‐of‐the‐art segmentation models (Vessel wall: $p\leq 6.0\times 10^{-6}$; Lumen: $p\leq 2.6\times 10^{-3}$; Outer wall: $p\leq 2.6\times 10^{-4}$) in comparison. We note that FGSM is equivalent to Model I in the ablation studies. Figure 5 compared manually delineated and the predicted lumen and outer wall contours for an example carotid artery.

**TABLE 2:** Comparison of the performance of proposed framework with those of existing noisy label learning approaches and the state‐of‐the‐art segmentation models.

	DSC(%)			HD95 (mm)		ASD (mm)	
Methods	Vessel Wall	Lumen	Outer Wall	Lumen	Outer Wall	Lumen	Outer Wall
Baseline	63.02 ± 25.74	79.92 ± 28.38	78.68 ± 29.25	1.59 ± 2.48	1.87 ± 2.59	0.74 ± 1.33	0.81 ± 1.09
Bootstrapping‐hard^18^	67.68 ± 14.13	86.13 ± 16.25	84.69 ± 15.61	2.19 ± 3.67	2.76 ± 4.01	0.88 ± 1.61	1.09 ± 1.58
Confidence penalty^11^	68.16 ± 17.03	84.06 ± 23.86	83.80 ± 21.04	1.32 ± 1.90	1.59 ± 1.85	0.55 ± 1.04	0.66 ± 0.75
Consistency regularization^12^	66.17 ± 22.40	85.03 ± 21.82	83.18 ± 23.32	1.06 ± 1.67	1.34 ± 1.66	0.42 ± 0.70	0.58 ± 0.68
Co‐teaching^31^	68.11 ± 17.20	85.63 ± 20.42	84.49 ± 19.63	1.61 ± 2.80	2.11 ± 3.28	0.67 ± 1.55	0.81 ± 1.18
FGSM^15^	68.54 ± 17.69	85.43 ± 20.23	84.94 ± 19.73	2.22 ± 3.82	2.34 ± 3.61	0.97 ± 1.91	1.03 ± 1.77
Tri‐teaching^32^	63.45 ± 22.61	82.00 ± 27.04	80.26 ± 26.27	1.29 ± 2.03	1.66 ± 2.07	0.54 ± 0.94	0.71 ± 0.93
VAT^33^	66.70 ± 17.24	85.03 ± 19.96	83.77 ± 19.41	1.77 ± 2.62	1.92 ± 2.33	0.72 ± 1.21	0.79 ± 0.91
UNet++ ^34^	62.92 ± 22.94	78.99 ± 27.83	78.87 ± 27.21	2.24 ± 3.47	2.39 ± 3.40	0.98 ± 1.71	1.06 ± 1.56
UNeTR^35^	56.44 ± 12.49	78.26 ± 17.01	74.35 ± 14.62	5.12 ± 4.76	6.02 ± 4.27	1.96 ± 1.80	2.35 ± 1.59
nnUNet^29^	65.21 ± 17.28	84.03 ± 23.05	82.44 ± 21.18	1.22 ± 1.54	1.68 ± 1.51	0.44 ± 0.50	0.62 ± 0.44
Ours	**73.51** ± **11.08**	**90.76** ± **10.21**	**90.10** ± **10.38**	**1.04** ± **1.44**	**1.25** ± **1.49**	**0.36** ± **0.48**	**0.51** ± **0.53**

*Note*: The best results are highlighted in bold.

Abbreviations: ASD, average surface distance; DSC, Dice similarity coefficients; FGSM, fast gradient sign method; HD95, Hausdorff distance.

The training time and inference time of the nine methods involved in the comparison are listed in Table 3. The inference time reported was the time required to segment an image volume with eight slices. Boostrapping‐hard and confidence penalty involve a single model and require a similar training time as the baseline model. Consistency regularization involves a single model but has two segmentation prediction branches for SNAP images that use different augmentation strategies. Thus, it requires a long training time than the bootstrapping‐hard and confidence penalty methods. Co‐teaching, VAT, FGSM, and our method each require two back‐propagation operations per training batch, resulting in longer training time than methods discussed above. In co‐teaching, two models were updated sequentially by back‐propagation within each training batch. VAT and FGSM, on the other hand, used a single model, but in addition to the need for a back‐propagation operation to update the model parameters, they require another back‐propagation operation to compute the gradient of the loss with respect to the original SNAP image map, which was subsequently used to generate the adversarial map. Our method also involves two models; however, back‐propagation was applied only to the student model, as the mean teacher model's parameters were updated using EMA. For this reason, the training time for our method is longer than that of consistency regularization but comparable to co‐teaching, VAT, and FGSM. Since segmentation models are trained offline, inference time is a more important consideration than training time for clinical applications. The differences in the inference time among the methods are small and would not have an impact on the clinical workflow.

**TABLE 3:** Training and inference times of methods involved in the comparison.

Methods	Total time (hrs)	Inference time (s)
Baseline	2.47	0.08
Bootstrapping‐hard^18^	3.06	0.09
Confidence penalty^11^	3.01	0.08
Consistency regularization^12^	3.56	0.08
Co‐teaching^31^	5.75	0.17
FGSM^15^	5.50	0.09
Tri‐teaching^32^	9.08	0.25
VAT^33^	5.42	0.08
UNet++ ^34^	2.69	0.17
UNeTR^35^	1.29	0.13
Ours	6.01	0.16

Abbreviation: FGSM, fast gradient sign method.

## 4 DISCUSSION

SNAP imaging inherently produces registered images with multiple contrasts, thereby enabling the characterization of various plaque components without requiring registering images with different contrasts as in conventional multi‐contrast MRI. However, training a deep learning approach for vessel wall segmentation from SNAP poses a challenge. Since SNAP is a relatively new imaging approach, manual segmentation required for training was performed in validated conventional MRI and then registered to the SNAP images. This process introduces the risk of misregistration, resulting in noisy labels that could degrade model performance if used directly for training. A straightforward solution to this issue would be to remove all misaligned images from the training set. However, our results showed that excluding misaligned images from training led to a significant drop in segmentation performance. This finding underscores the importance of developing models that incorporate regularization and label correction techniques to effectively utilize noisy training data, such as the misaligned images in our application. This study proposes a novel approach that leverages misaligned (or noisy) boundaries to enhance segmentation performance.

The proposed segmentation framework demonstrated statistically significant performance improvements over seven existing segmentation models developed to handle noisy labels. Our contribution involves integrating adversarial training with an approach to correct the label for improved training guidance based on misaligned training images. Our label correction approach was inspired by Reed et al.,^18^ in which they added the noisy label with the model's prediction, referred to as the pseudo‐label, to generate an improved training target. Training on the improved target mitigated the effect of noisy labels on classification performance. The pseudo‐label applied was hardened before adding it with the noisy label. In their approach, the pseudo‐label was transformed into a hardened output by assigning a probability of 1 to the class with the highest predicted probability while setting all other class probabilities to 0. Their experiments showed that hardening the pseudo‐label before addition with the noisy label improved the accuracy in emotion classification based on facial images.

We developed the first segmentation framework that utilized this label improvement strategy, but unlike Reed et al.,^18^ we integrated the student and the mean teacher predictions to generate the pseudo‐label and we implemented a selective strategy to harden the pseudo‐label. In our approach, the probability at each pixel is hardened only if the student and mean teacher models reach a consensus on the class with the highest predicted probability. Furthermore, probability hardening is only performed if the highest predicted probabilities from both the student and mean teacher models exceed a threshold τ. The parameter τ varies from 0.33 to 1 in our parameter tuning experiment. The setting τ=0.33 represents the case where probability hardening is performed without a requirement of a lower limit in the probability because, as a three‐class problem, the class with the highest predicted probability must have a probability larger than 1/3. On the other end of the spectrum, τ=1 represents the case where there is no probability hardening. The result presented in Figure 6 shows that probability hardening without enforcing a lower limit on predicted probability (i.e., τ=0.33) does not improve segmentation performance compared to the case with no probability hardening (i.e., τ=1). This result contrasts with the finding in Reed et al.^18^ in the context of their classification problem. However, our innovation of selective hardening with a range of τ from 0.4 to 0.99 all contribute to improved $DSC_{avg}$. We note that the optimized τ is high at 0.99. As illustrated in Figure 4, the student and the mean teacher frameworks provided a correct prediction with a high probability for regions away from the vessel wall boundaries, but the probability may not be as high as 1. Probability hardening in these regions provided more confident masks for the optimization in Path 2 and led to better segmentation performance.

Notably, Path 1 and Path 2 of our model were driven by the multi‐class Dice loss and the weighted CE loss, respectively. This choice stems from the nature of their segmentation labels: in Path 1, predictions were compared to binary manual segmentations, while in Path 2, they were evaluated against a surrogate ground truth, a soft label with continuous pixel values between 0 and 1. A loss function is expected to be minimized when the predicted segmentation matches the label segmentation exactly. However, this expectation does not hold for Dice loss in the presence of a soft label. Instead, the Dice loss pushes predictions towards extreme values (i.e., 0 or 1) rather than matching the soft label distribution.^36^, ^37^, ^38^ In contrast, the CE loss optimizes probability distribution alignment with the loss minimized when the prediction matches the soft segmentation label.^39^ For this reason, the CE loss was chosen to optimize the prediction in Path 2.

Although the proposed segmentation approach attained high accuracy in segmenting the carotid vessel wall from SNAP images, the study is limited to a single SNAP imaging dataset from one institute. This study is designed to demonstrate that the carotid vessel wall can be directly segmented from SNAP imaging sequences. Before this hypothesis is validated, vessel wall contours segmented from conventional multi‐contrast images must be used as training labels. The workflow involved in this study, which pilots direct segmentation from SNAP images, is time‐consuming, making it challenging to further scale up the study. It involves the acquisition of conventional multi‐contrast and SNAP images, manual segmentation from the multi‐contrast images and the registration of segmented contours to SNAP images. Nonetheless, the diversity of the dataset used in this pilot study remains crucial for demonstrating the generalizability of our approach. We note that the arterial conditions of patients in this data set are highly diverse, as detailed in Zhang et al.^5^ in terms of age, sex, percent stenosis (11.9±25.2%), American Heart Association plaque type (from Type I to VIII) and plaque components identified. The dataset encompasses arterial images with intraplaque hemorrhage, lipid‐rich/necrotic core and calcification identified. These components exhibit highly variable SNAP intensity characteristics, leading to substantial variation in the contrast among the vessel wall, the lumen, and the background across different arteries. With this study confirming our hypothesis that the vessel wall can be directly segmented from SNAP images, the segmentation workflow can be significantly streamlined in future studies, as the conventional multi‐contrast images are no longer needed. Under this streamlined framework involving only the SNAP images, the generalizability of the proposed method should be further evaluated in a future study with SNAP images acquired from multiple centers that encompass patient populations with varying degrees of plaque burden (from minimal to severe) and diverse plaque types.

Second, our approach requires manual identification of misaligned segmentation. This requirement did not create a bottleneck in our study, as manual identification was available through prior work on plaque components,^5^ where misaligned images were excluded from their analysis. Automated methods for labeling misaligned segmentation can be introduced to address this limitation. For example, signal contrast along segmented boundaries^40^ could be leveraged to develop an automated labeling approach, improving reproducibility and reducing user interaction. Although our approach allows direct vessel wall segmentation from SNAP images and eliminates the need for registration from conventional multi‐contrast MRI, automated labeling methods remain valuable for validating new MR imaging sequences, which requires registration of segmented boundaries from established MR imaging modalities.

## 5 CONCLUSION

We, for the first time, develop an approach that can reliably segment the carotid vessel walls directly from SNAP images. As SNAP imaging has not been shown previously to be capable of identifying vessel wall, reliable vessel wall boundaries required to train segmentation models were segmented from conventional multi‐contrast MRI and registered to SNAP images. However, misregistration can produce misaligned (or noisy) boundaries, which, if directly used in training, would reduce segmentation performance. The proposed model addresses this issue by integrating adversarial training with a novel label correction strategy involving (i) the convex combination of pseudo‐labels and misaligned manual segmentation and (ii) a label hardening technique. This proposed framework enables direct vessel wall segmentation from SNAP images before plaque component analysis. As SNAP imaging offers greater longitudinal coverage of the carotid artery and reduced slice thickness compared to conventional multi‐contrast MRI, the proposed approach facilitates more detailed plaque component analysis over a longer arterial segment.

## CONFLICT OF INTEREST STATEMENT

The authors declare no conflicts of interest.

## MODIFIED 3D U‐NET

We implemented several modifications to the 3D U‐Net backbone to better suit our application, as illustrated in Figure A.1. First, a small batch size was chosen to emphasize hard examples during training, but this disrupted batch normalization,^41^ necessitating the use of group normalization instead. Second, although 3D networks can model inter‐slice continuity, they have a larger number of trainable parameters and require a larger volume set to avoid overfitting.^42^ To mitigate this, slice‐based convolutions were applied in most layers, except near the bottleneck, to preserve longitudinal continuity of the carotid artery. Additionally, a 1×5×5 convolution, group normalization, and leaky ReLU layers were added at the input to expand the receptive field. Three‐dimensional dropout was introduced before the first up‐convolution and output layers to enhance regularization.

## References

[1] Saba L , Yuan C , Hatsukami TS , et al. Carotid artery wall imaging: perspective and guidelines from the asnr vessel wall imaging study group and expert consensus recommendations of the american society of neuroradiology. Am J Neuroradiol. 2018;39:9‐31. 10.3174/ajnr.A5488 PMC7410574 29326139

[2] Ooi YC , Gonzalez NR , Management of extracranial carotid artery diseasen. Cardiol Clin. 2015;33:1‐35. 25439328 10.1016/j.ccl.2014.09.001 PMC4694631

[3] Wang J , Börnert P , Zhao H , et al. Simultaneous noncontrast angiography and intraPlaque hemorrhage (SNAP) imaging for carotid atherosclerotic disease evaluation. Magn Reson Med. 2013;69:337‐345. 22442116 10.1002/mrm.24254 PMC3418400

[4] Chen S , Zhao H , Li J , et al. Evaluation of carotid atherosclerotic plaque surface characteristics utilizing simultaneous noncontrast angiography and intraplaque hemorrhage (SNAP) technique. J Magn Reson Imaging. 2018;47:634‐639. 28766810 10.1002/jmri.25815 PMC5796877

[5] Zhang Q , Qiao H , Dou J , et al. Plaque components segmentation in carotid artery on simultaneous non‐contrast angiography and intraplaque hemorrhage imaging using machine learning. Magn Reson Imaging. 2019;60:93‐100. 30959178 10.1016/j.mri.2019.04.001

[6] Jiang M , Spence JD , Chiu B . Segmentation of 3D ultrasound carotid vessel wall using U‐Net and segmentation average network. In: 42nd Annual International Conference of the IEEE Engineering in Medicine & Biology Society, EMBC 2020, Montréal, QC, Canada, July 20‐24, 2020 . IEEE; 2020:2043‐2046. 10.1109/EMBC44109.2020.9175975 33018406

[7] Zhou R , Guo F , Azarpazhooh MR , et al. A voxel‐based fully convolution network and continuous max‐flow for carotid vessel‐wall‐volume segmentation from 3D ultrasound images. IEEE Trans Med Imaging. 2020;39:2844‐2855. 32142426 10.1109/TMI.2020.2975231

[8] Wu J , Xin J , Yang X , et al. Deep morphology aided diagnosis network for segmentation of carotid artery vessel wall and diagnosis of carotid atherosclerosis on black‐blood vessel wall MRI. Med Phys. 2019;46:5544‐5561. 31356693 10.1002/mp.13739

[9] Zhang C , Bengio S , Hardt M , Recht B , Vinyals O . Understanding deep learning requires rethinking generalization. Commun ACM. 2016;64:107‐115.

[10] Arpit D , Jastrzundefinedbski S , Ballas N , et al. A closer look at memorization in deep networks. In: Precup D , Teh YW , eds., PMLR. Proceedings of the 34th International Conference on Machine Learning . 2017;70:233‐242.

[11] Pereyra G , Tucker G , Chorowski J , Kaiser L , Hinton GE . Regularizing neural networks by penalizing confident output distributions. In: *5th International Conference on Learning Representations, ICLR 2017, Toulon, France, April 24‐26, 2017, Workshop Track Proceedings*, OpenReview.net; 2017.

[12] Luo Y , Zhu J , Pfister T . A simple yet effective baseline for robust deep learning with noisy labels. arXiv preprint arXiv:1909.09338 (2019).

[13] Song H , Kim M , Park D , Shin Y , Lee J‐G . Learning from noisy labels with deep neural networks: A survey. IEEE Transactions on Neural Networks and Learning Systems. 2023;34:8135‐8153. 10.1109/TNNLS.2022.3152527 35254993

[14] Göçeri E . Medical image data augmentation: techniques, comparisons and interpretations. Artif Intell Rev. 2023;56:12561‐12605. 10.1007/s10462-023-10453-z PMC10027281 37362888

[15] Goodfellow IJ , Shlens J , Szegedy C . Explaining and harnessing adversarial examples. In: Bengio Y , LeCun Y , eds., 3rd International Conference on Learning Representations, ICLR 2015, San Diego, CA, USA, May 7‐9, 2015, Conference Track Proceedings . 2015.

[16] Fatras K , Damodaran BB , Lobry S , Flamary R , Tuia D , Courty N . Wasserstein adversarial regularization for learning with label noise. IEEE Trans Pattern Anal Mach Intell. 2022;44:7296‐7306. 34232864 10.1109/TPAMI.2021.3094662

[17] Tanaka D , Ikami D , Yamasaki T , Aizawa K . Joint optimization framework for learning with noisy labels. In: 2018 IEEE Conference on Computer Vision and Pattern Recognition, CVPR 2018, Salt Lake City, UT, USA, June 18‐22, 2018 . IEEE Computer Society; 2018:5552‐5560.

[18] Reed S , Lee H , Anguelov D , Szegedy C , Erhan D , Rabinovich A . Training deep neural networks on noisy labels with bootstrapping. In: Bengio Y , LeCun Y , eds., 3rd International Conference on Learning Representations, ICLR 2015,Workshop Track Proceedings ; 2015.

[19] Han J , Luo P , Wang X . Deep self‐learning from noisy labels. In: *2019 IEEE/CVF International Conference on Computer Vision, ICCV 2019, Seoul, Korea (South), October 27 ‐ November 2, 2019*, Los Alamitos, CA, USA. IEEE Computer Society; 2019:5137‐5146.

[20] Lee DH . Pseudo‐Label: The simple and efficient semi‐supervised learning method for deep neural networks. In: WREPL. 2013;3:896.

[21] Chen B , Jiang J , Wang X , Wan P , Wang J , Long M . Debiased self‐training for semi‐supervised learning. In: Koyejo S , Mohamed S , Agarwal A , Belgrave D , Cho K , Oh A eds. Advances in Neural Information Processing Systems 35: Annual Conference on Neural Information Processing Systems 2022, NeurIPS 2022, New Orleans, LA, USA, November 28 ‐ December 9, 2022 ; 2022.

[22] Nguyen DT , Mummadi CK , Ngo T , Nguyen THP , Beggel L , Brox T . SELF: Learning to filter noisy labels with self‐ensembling. In: 8th International Conference on Learning Representations, ICLR 2020, Addis Ababa, Ethiopia, April 26‐30, 2020 ; 2020.

[23] Liu S , Zhi S , Johns E , Davison AJ . Bootstrapping semantic segmentation with regional contrast. In: *The Tenth International Conference on Learning Representations, ICLR 2022, Virtual Event, April 25‐29, 2022*. OpenReview.net; 2022.

[24] Tarvainen A , Valpola H . Mean teachers are better role models: Weight‐averaged consistency targets improve semi‐supervised deep learning results. In: Guyon I , von Luxburg U , Bengio S , Wallach HM , Fergus R , Vishwanathan SVN , Garnett R , eds. *Advances in Neural Information Processing Systems 30: Annual Conference on Neural Information Processing Systems 2017, December 4‐9, 2017, Long Beach, CA, USA*. arXiv; 2017:1195‐1204.

[25] Yuan C , Kerwin WS , Yarnykh VL , et al. MRI of atherosclerosis in clinical trials. NMR Biomed. 2006;19:636‐654. 16986119 10.1002/nbm.1065

[26] Song H , Kim M , Lee JG . SELFIE: Refurbishing unclean samples for robust deep learning. In: Chaudhuri K , Salakhutdinov R eds. Proceedings of the 36th International Conference on Machine Learning , vol. 97 of *Proceedings of Machine Learning Research*. PMLR; 2019:5907‐5915.

[27] Yi K , Wu J . Probabilistic end‐to‐end noise correction for learning with noisy labels. In: IEEE Conference on Computer Vision and Pattern Recognition, CVPR 2019, Long Beach, CA, USA, June 16‐20, 2019 . IEEE; 2019:7017‐7025.

[28] Tukey JW . Comparing individual means in the analysis of variance. Biometrics. 1949;5:99‐114. 18151955

[29] Isensee F , Jaeger PF , Kohl AAS , Petersen J , Maier‐Hein KH . nnU‐Net: a self‐configuring method for deep learning‐based biomedical image segmentation. Nat Methods. 2020;18:203‐211. 33288961 10.1038/s41592-020-01008-z

[30] Kingma DP , Ba J . Adam: A method for stochastic optimization. In: Bengio Y , LeCun Y eds. 3rd International Conference on Learning Representations, ICLR 2015, San Diego, CA, USA, May 7‐9, 2015, Conference Track Proceedings ; 2015.

[31] Han B , Yao Q , Yu X , Niu G , et al. Co‐teaching: Robust training of deep neural networks with extremely noisy labels. In: Bengio S , Wallach HM , Larochelle H , Grauman K , Cesa‐Bianchi N , Garnett R eds. *Advances in Neural Information Processing Systems 31: Annual Conference on Neural Information Processing Systems 2018, NeurIPS 2018, December 3‐8, 2018, Montréal, Canada*. ; 2018:8536‐8546.

[32] Xue C , Deng Q , Li X , Dou Q , Heng P‐A . Cascaded robust learning at imperfect labels for chest X‐ray segmentation. In: Medical Image Computing and Computer Assisted Intervention – MICCAI 2020. Springer International Publishing; 2020: pp. 579‐588

[33] Miyato T , Maeda Si , Koyama M , Ishii S . Virtual adversarial training: a regularization method for supervised and semi‐supervised learning. IEEE Trans Pattern Anal Mach Intell. 2017;41:1979‐1993. 10.1109/TPAMI.2018.2858821 30040630

[34] Zhou Z , Siddiquee MMR , Tajbakhsh N , Liang J . UNet++: A nested U‐Net architecture for medical image segmentation. In: Stoyanov D , Taylor Z , Carneiro G , Syeda‐Mahmood TF , Martel AL , Maier‐Hein L , Tavares JMRS , Bradley AP , Papa JP , Belagiannis V , Nascimento JC , Lu Z , Conjeti S , Moradi M , Greenspan H , Madabhushi A eds. Deep Learning in Medical Image Analysis ‐ and ‐ Multimodal Learning for Clinical Decision Support ‐ 4th International Workshop, DLMIA 2018, and 8th International Workshop, ML‐CDS 2018, Held in Conjunction with MICCAI 2018, Granada, Spain, September 20, 2018 , vol. 11045. Springer; 2018:3‐11. 10.1007/978-3-030-00889-5_1 PMC7329239 32613207

[35] Hatamizadeh A , Tang Y , Nath V , et al. UNETR: Transformers for 3D Medical Image Segmentation. In: IEEE/CVF Winter Conference on Applications of Computer Vision, WACV 2022, Waikoloa, HI, USA, January 3‐8, 2022 , IEEE; 2022:1748‐1758.

[36] Wang Z , Popordanoska T , Bertels J , Lemmens R , Blaschko MB . Dice semimetric losses: optimizing the dice score with soft labels. In: Greenspan H , Madabhushi A , Mousavi P , Salcudean S , Duncan J , Syeda‐Mahmood T , Taylor R eds. Medical Image Computing and Computer Assisted Intervention – MICCAI 2023, Springer Nature Switzerland; 2023:475‐485.

[37] Bertels J , Robben D , Vandermeulen D , P,. Suetens. Theoretical analysis and experimental validation of volume bias of soft dice optimized segmentation maps in the context of inherent uncertainty. Med Image Anal. 2021;67:101833. 33075643 10.1016/j.media.2020.101833

[38] Nordströ m M , Hult H , Maki A , Löfman F . Noisy image segmentation with soft‐dice. arXiv preprint arXiv:2304.00801 (2023).

[39] Hinton G , Vinyals O , Dean J . Distilling the Knowledge in a neural network. arXiv preprint arXiv:1503.02531 (2015).

[40] Chiu B , Krasinski A , Spence JD , Parraga G , Fenster A . Three‐dimensional carotid ultrasound segmentation variability dependence on signal difference and boundary orientation. Ultrasound Med Biol. 2010;36:95‐110. 19900751 10.1016/j.ultrasmedbio.2009.08.005

[41] Wu Y , He K . Group normalization. In: Ferrari V , Hebert M , Sminchisescu C , Weiss Y eds. Computer Vision – ECCV 2018, Lecture Notes in Computer Science, vol 11217. Springer; 2018:3‐19.

[42] Zettler N , Mastmeyer A . Comparison of 2D vs 3D U‐Net organ segmentation in abdominal 3D CT images. arXiv preprint arXiv:2107.04062 ; 2021.

